# APOE Polymorphism Is Associated with Changes in the Kynurenine Pathway

**DOI:** 10.3390/genes14101955

**Published:** 2023-10-18

**Authors:** Per G. Farup, Helge Rootwelt, Knut Hestad

**Affiliations:** 1 Department of Research, Innlandet Hospital Trust, 2381 Brumunddal, Norway; knut.hestad@ntnu.no; 2 Department of Clinical and Molecular Medicine, Faculty of Medicine and Health Sciences, Norwegian University of Science and Technology, 7491 Trondheim, Norway; 3 Department of Medical Biochemistry, Oslo University Hospital, 0424 Oslo, Norway; helge.rootwelt@ous-hf.no; 4 Department of Psychology, Faculty of Social and Educational Sciences, Norwegian University of Science and Technology, 7491 Trondheim, Norway

**Keywords:** APOE, kynurenine pathway, obesity, depression, neurological symptoms, quinolinic acid, kynurenine, inflammation

## Abstract

Background: APOE polymorphism and the Kynurenine pathway (KP) are associated with many disorders, but little is known about associations between APOE polymorphism and the KP. This study explored the associations between the KP and APOE polymorphism in disorders associated with APOE polymorphism and changes in the KP. Methods: Subjects with morbid obesity before and after bariatric surgery (numbers 139 and 95, respectively), depression (number 49), and unspecified neurological symptoms (number 39) were included. The following grouping of the APOE genotypes was used: E2 = ɛ2ɛ2 + ɛ2ɛ3, E3 = ɛ3ɛ3 + ɛ2ɛ4, and E4 = ɛ3ɛ4 + ɛ4ɛ4. The KP metabolites Tryptophan, Kynurenine, Kynurenic acid, Quinolinic acid, and Xanthurenic acid were quantified in serum. Results: The main findings were a significant positive association between E3 and Quinolinic acid (difference between E3 and E2E4: 12.0 (3.5; 18.6) ng/mL); *p* = 0.005), and a negative association between E4 and Kynurenine (difference between E4 and E2E3: −31.3 (−54.2; −3.2) ng/mL; *p* = 0.008). Quinolinic acid has been ascribed neurotoxic and inflammatory effects, and Kynurenine is a marker of inflammation. Conclusions: The findings indicate that APOE polymorphism might cause changes in the KP that contribute to the disease. Inflammation could be the link between APOE and the KP.

## 1. Introduction

Tryptophan, which is involved in a wide range of benign and malignant disorders, is metabolised by three pathways: The Kynurenine pathway (KP), with degradation mainly in the liver, is associated with neuroactive and immunomodulatory effects; the Serotonin pathway, with degradation in the gut, is associated with behavioural and neuroendocrine functions; and the Indole-Pyruvate pathway, with degradation by the gut microbiota, is associated with intestinal physiology. The interplay between the pathways is complex; all pathways might be involved in one disease [1].

The KP, the main pathway, is involved in the pathophysiology of disorders in the central nervous system and other organ systems and body functions. Examples are mental health disorders (dementia, anxiety, and depression), endocrine diseases (diabetes, insulin resistance), diseases of the liver and pancreas, energy homeostasis (e.g., obesity, thermogenesis), skeletal muscle functions, metabolic disorders (cardiovascular diseases and atherosclerosis), pulmonary diseases, and carcinogenesis [2,3,4,5,6,7,8,9,10]. Activation of the immune system is a common feature of the disorders associated with changes in the KP [2,4,5,11]. The association between the KP and the immune system is clearly seen in subjects with obesity, metabolic/endocrine and psychiatric disease [5,6,11]. Although causal relationships between the KP and diseases are uncertain, modulation of the KP by enzyme inhibitors or KP metabolites has been proposed for the prevention and treatment of a range of both benign and malignant diseases, such as neurological, psychiatric, metabolic, and intestinal diseases [2,3,12].

Genetic APOE polymorphism is associated with an equally wide range of diseases. Lumsden et al. have written a comprehensive overview [13]. Compared with the genotype ɛ3ɛ3, the ɛ2ɛ3 and ɛ2ɛ4 genotypes are associated with reduced hyperlipidemia and ischemic cardiovascular diseases, and the ɛ2ɛ2 genotype with an increase in peripherical vascular diseases (thrombosis and embolism) and familial dysbetalipoproteinemia [13,14]. ɛ3ɛ4 and ɛ4ɛ4 are associated with an increase in all types of dementia, mental and neurological disorders, hyperlipidemia, and ischemic cardiovascular diseases, but with a reduction in obesity and chronic obstructive pulmonary diseases [13].

Despite the comprehensive literature on the associations between the KP and diseases, and between APOE polymorphism and diseases, there are only a few studies on the associations between APOE polymorphism and the KP. The studies we know of focus on the ɛ4 allele and are limited to subjects with dementia [15,16,17,18]. They showed no significant associations between the ɛ4 allele and the KP. The lack of established associations between ɛ4 and the KP in subjects with dementia does not exclude associations between APOE polymorphism and the KP in subjects with other disorders known to be associated with both APOE polymorphism and changes in the KP.

This study used data from two clinical studies including subjects with morbid obesity, depression, and unspecified neurological symptoms [19,20]. These disorders are associated with changes in the KP and APOE polymorphism which make them appropriate for the study of associations between the KP and APOE polymorphism. Previous papers have shown that the levels of KP metabolites differ between the groups [21,22]. Our assumption is that the overall effects of the APOE polymorphism on the KP are similar in the disease groups.

This study explored the overall associations between the KP and APOE polymorphism in subjects with morbid obesity, depression, and unspecified neurological symptoms. Observed associations could indicate that changes in the KP are genetically mediated.

## 2. Materials and Methods

### 2.1. Data and Study Design

The study retrospectively used data from two clinical studies at Innlandet Hospital Trust, Brumunddal, Norway. Data from the prospective cohort study Morbid Obesity—Bio-Psycho-Social impacts (MO-BiPS) were combined with data from the cross-sectional study Depression-Immunology [19,20].

The MO-BiPS study included consecutive subjects with morbid obesity (defined as BMI > 40 kg/m^2^ or BMI > 35 kg/m^2^ with obesity-related comorbidity) referred to the Obesity Unit at Innlandet Hospital Trust for evaluation of bariatric surgery. Subjects with severe not obesity-related comorbidity were excluded. After inclusion and before surgery, the subjects followed a strict program with regular follow-ups for six months with information about obesity, bariatric surgery, dietary recommendations, and physical activity. There were regular follow-ups after surgery. This study used data from inclusion in the study and six months after surgery.

The depression group comprised participants with a diagnosis of depression according to the International Classification of Diseases, tenth edition (F32–34 spectra), recruited from the Mental Health Unit at Innlandet Hospital Trust. The control group comprised patients with neurological symptoms but no neurological signs or laboratory findings indicating neurological disease or disorder, referred to the Department of Neurology at the same hospital. A complete description is given in the paper by Hestad et al. [22].

### 2.2. Variables

The following variables from both studies were used: Age (years), sex (biological), body mass index (BMI: kg/m^2^), daily smoking (no/yes), diabetes (no/yes) and C-reactive protein (CRP: mg/L).

The KP metabolites (Tryptophan (Trypt), Kynurenine (Kyn), Kynurenic acid (KA), Quinolinic acid (QA), and Xanthurenic acid (XA)) were quantified in serum and the ratios Kyn/Trypt (K/T ratio × 1000), KA/Kyn × 1000, KA/QA × 1000, KA/XA, and QA/XA were calculated. The metabolites were reported as ng/mL. After adding 20 µL internal standard solution (one deuterated substance for each of the analytes), protein precipitation of 20 µL human plasma was performed using 60 µL 50% (*w*/*v*) trichloroacetic acid (TCA) in water. An aliquot of 5 µL was injected from the supernatant into the high-performance liquid chromatography (HPLC) system after thorough mixing for 8 min and centrifugation for 15 min (4000× *g* at 20 °C). HPLC was performed with an Agilent 1260 Infinity liquid chromatograph (Agilent Technologies, Palo Alto, CA, USA) with an Agilent 6460 Triple Quad LC/MS detector. Separation of Trypt and the metabolites was performed on a 3.0 mm × 100 mm, 2.6 µm, EVO C18 reversed-phase column from Phenomenex (Phenomenex Inc., Torrance, CA, USA). The column temperature was 40 °C. The gradient was from 100% 0.1% formic acid in water to 100% 0.1% formic acid in acetonitrile. To quantify Trypt and the metabolites in test samples and control samples, a seven-point calibration curve was created for each analyte. The KP analyses were performed by VITAS AS, Oslo, Norway.

The Department of Medical Biochemistry, Oslo University Hospital—Rikshospitalet, Oslo, Norway, performed the genotyping using allele-specific real-time polymerase chain reactions. In accordance with other papers, e.g., the review by Lumsden et al., the following order of the APOE genotypes was used: ɛ2ɛ2, ɛ2ɛ3, ɛ3ɛ3, ɛ2ɛ4, ɛ3ɛ4, ɛ4ɛ4 [13]. There is no accepted grouping of the genotypes. Based on the order of the APOE variants and because some groups were small, we combined the genetic variants into three groups referred to as the E-groups: E2 = ɛ2ɛ2 + ɛ2ɛ3, E3 = ɛ3ɛ3 + ɛ2ɛ4, and E4 = ɛ3ɛ4 + ɛ4ɛ4 according to Han et al. [23]. The grouping of the ɛ2ɛ4 genotype into the E-groups is not obvious. However, the paper by Lundsten et al., including 337,484 subjects, reports differences between ɛ3ɛ3 and ɛ2ɛ4 in more than 950 disease outcomes, of which only three (disorders of lipid metabolism, hyperlipidemia, and hypercholesterolemia) were statistically significant after adjusting for multiple testing [13]. The paper thereby documents the minor differences between ɛ3ɛ3 and ɛ2ɛ4 and is the reason for including ɛ2ɛ4 together with ɛ3ɛ3 in the E3 group. The combination of two E-groups, e.g., E2 and E3, was abbreviated E2E3, not to be mistaken for the genotype ɛ2ɛ3.

### 2.3. Statistics

The results are given as mean (SD), median (min; max) and number (proportion in percentage). Some results are given both as mean (SD) and median (min; max) to present deviations from normal distributions. All analyses were performed on original data, and some analyses were also performed on logarithmic (ln) transformed data because of deviations from a normal distribution. The levels of the KP metabolites in the E-groups and comparisons between the E-groups were analysed with a linear mixed model and reported as estimated marginal means with 95% confidence intervals (CI) and *p*-values. To account for the KP-metabolites’ deviations from a normal distribution, the pairwise comparisons between the E-groups were analysed with a linear mixed model based on 10,000 bootstrap samples and reported as the differences between the mean values with a 95%CI (percentile) and *p*-values. Statistical analyses were performed with IBM Corp. Released 2020: IBM SPSS Statistics for Windows, Version 27.0. Armonk, NY, USA: IBM Corp. To reduce the possibility of type 1 errors, *p* ≤ 0.01 was chosen as statistically significant in this explorative study with multiple tests.

## 3. Results

### 3.1. Subject Characteristics and Material

The study included 219 subjects: 139 with morbid obesity, of whom 95 had a follow-up six months after bariatric surgery, 49 with depression, and 31 with unexplained neurological symptoms. Table 1 gives the subject characteristics.

The amount of KP metabolites in serum is given in Table 2. There were differences between the diseases and between before and after bariatric surgery. Some of the differences have been shown in previous studies [21].

### 3.2. Overall Effects

The concentrations of the KP metabolites are presented for each of the E-groups. Because the data deviated from a normal distribution, comparisons between the groups were performed on original and ln-transformed data. The results based on original and ln-transformed data were similar and showed no significant differences between the E-groups. Although not significant, the QA estimates for E2 and E4 were similar and lower than E3, and the Kyn estimates for E2 and E3 were of the same order and higher than E4. Table 3 gives the results and the comparisons between the groups based on the original data.

### 3.3. Pairwise Analyses

Analyses were performed to detect differences in the KP metabolites between pairs of the E-groups. Pairwise analyses between one and one of the E-groups were not performed because the overall analyses in Table 3 showed no significant differences between the groups. Pairwise comparisons between one E-group and the combination of the two other E-groups were performed because such differences have been presented, particularly for the E4 group, because our results (Table 3) indicated such differences, and because the increase in group size provides higher statistical power. The pairwise comparisons were performed on original data with a linear mixed model and bootstrapping and reported as differences between the means with 95% CIs (percentiles) and *p*-values. The main findings were the significantly elevated level of QA in the E3 group compared with the E2E4 group and the low level of Kyn in the E4 group compared with the E2E3 group. Table 4 gives all the results.

### 3.4. Fully Adjusted Post Hoc Analyses

To further explore the significant associations between Kyn and E4 and between QA and E3, the analyses were adjusted for BMI, diabetes, smoking, and CRP in addition to age, sex, diagnosis, and point of time (before and after bariatric surgery). These analyses did not affect the significant associations between QA and E3. However, the estimated difference in Kyn between E4 and E2E3 was markedly reduced and was no longer statistically significant. Table 5 gives the details.

## 4. Discussion

In this study, the new findings were the positive associations between E3 and QA and QA/XA ratio, and the negative association between E4 and Kyn. The analyses were adjusted for age, sex, diagnosis, and point of time (before and after surgery for the obesity group). The association between E3 and QA did not change when adjusting for diabetes, BMI, CRP, and smoking, indicating a direct effect of E3 on the KP or an effect via other mediators. The reduced difference in Kyn between E4 and E2E3 and the lack of significance when adjusting for the same covariates indicated mediated indirect effects. The positive associations between the KP metabolites and age are known age-related changes in the biomarkers [24].

High QA levels have clinical implications. QA has been ascribed neurotoxic and inflammatory effects and is associated with delirium, mood disorders (anxiety and depression), neurodegenerative diseases (Alzheimer’s disease, amyotrophic lateral sclerosis, Parkinson’s disease), and cognitive decline [25,26,27,28]. ɛ4 has been associated with the same disorders, particularly dementia and cognitive impairment [13]. The positive association between E3 and QA was, therefore, unexpected. However, several studies have indicated an age-related effect of ɛ4 with unfavourable effects in old age and favourable effects in midlife, particularly in men, an example of antagonistic pleiotropy (i.e., genes differentially impact functions during the lifespan) [29,30,31]. The unfavourable effect of E3 with high QA in this study in middle-aged subjects is in accordance with the reported antagonistic pleiotropy.

E3, which was associated with high QA values, has been associated with a high prevalence of some disorders and protection against others. ɛ3ɛ3 has been associated with a high prevalence of obesity and pulmonary diseases (compared with ɛ4ɛ4), with diabetes type 2 and liver-biliary diseases (compared with ɛ3ɛ4), with unfavourable lipid markers and cardio-vascular diseases (compared with ɛ2ɛ3), and with reduced cognitive functions in adults less than 65 years of age (compared with ɛ4 and ɛ2) [13,29,32]. Our findings suggest that these disorders with a high prevalence in subjects with E3 might be due to elevated QA levels in subjects with E3. Since the association between QA and E3 was unaffected by adjusting for covariates assumed to be mediators of the effect, the association could be a direct effect of E3 on the QA metabolism or an indirect effect via unknown mediators [21]. A possible clinical consequence is to find new therapeutic interventions for the reduction of QA in subjects with E3 and diseases associated with E3.

Inflammation shifts the Trypt metabolism towards the KP and increases the amounts of Kyn [2,4,5,11]. The low Kyn level associated with E4 indicates reduced inflammation in subjects with E4. This is in accordance with the low prevalence of obesity and low CRP in the E4 group, since CRP, obviously, and obesity are also associated with inflammation [7,13]. Including covariates associated with inflammation (BMI, diabetes, age) in the multivariable analyses (Table 5) eliminated the statistically significant association between E4 and Kyn, indicating that these variables mediate the association. Although the covariates did not change the significant association between E3 and QA, it is not excluded that this association is also a marker of inflammation since both QA and the QA/XA ratio are markers of inflammation [21].

Most studies, like this one, of the Trypt metabolism include only one of the three Trypt metabolism pathways. All pathways (the KP, the Serotonin pathway, and the Indole-Pyruvate pathway) might be involved in the pathogenesis of one disease, and the interaction between the pathways might be as important as the changes in one pathway.

### Strengths and Limitations

The heterogeneity of the participating group (subjects with obesity, depression, and neurological symptoms) was both a strength and a limitation. The groups were judged too small to study the divergent effects of APOE polymorphism in the participating groups. On the other hand, the heterogeneity allowed the study of an overall, general association between APOE polymorphism and the KP, which was the aim of the study. The combination of the ɛ-groups, such as ɛ3ɛ3 + ɛ2ɛ4 into E3, is a weakness. The genotypes sometimes have different phenotypes, and the associations between the genotypes and the KP might differ. To reduce type 1 errors, the significance value was set to *p* ≤ 0.01. Nevertheless, the exploratory design with multiple tests does not exclude type 1 errors. Type 2 errors, mainly related to the low number of participants in the E2 group (*n* = 18), are not unlikely. The selection and registration of variables were similar in the two studies, which made the studies fit for merging. The KP analyses were performed at the same time in the same laboratory.

## 5. Conclusions

APOE polymorphism and KP abnormalities are associated with a variety of diseases and disorders, but studies on the associations between APOE polymorphism and KP abnormalities are limited. This study showed significant associations between E3 and high QA and QA/XA-ratios, and between E4 and low Kyn. The findings indicate that APOE polymorphism might cause changes in the KP that contribute to disease and disorders. Some, and perhaps most, of the changes in the KP might be related to inflammation. The effects are apparently different in middle-aged and elderly subjects (antagonistic pleiotropy).

## Figures and Tables

**Table 1:** Subject characteristics of the 219 participants are given as numbers (proportion in %) and mean (SD).

Diagnosis	Sex Female Male	Age Years	BMI Kg/m^2^	Smoking Yes	Diabetes Yes	CRP mg/L	APOE ɛ-Alleles 2.2/2.3/3.3/ 2.4/3.4/4.4
Obesity (before surgery, *n* = 139)	108 (78%) 31 (22%)	43.0 (8.7)	42.1 (3.8)	25 (18%)	26 (19%)	6.8 (5.7)	1(0.7%)/13(9%)/70(50%)/ 4(3%)/47(34%)/4(3%)
Obesity (after surgery, *n* = 95)	77 (81%) 18 (19%)	44.0 (8.2)	30.3 (3.7)	7 (7.4%)	17 (18%)	1.9 (2.5)	0 (0%)/11(12%)/49(52%)/ 2(2%)/31(33%)/2(2%)
Depression (*n* = 49)	26 (53%) 23 (47%)	45.3 (14.6)	25.8 (4.4)	23 (47%)	2 (4%)	2.1 (2.6)	0(0%)/3(6%)/29(59%)/ 0(0%)/15(31%)/2(4%)
Neurological (*n* = 31)	22 (71%) 9 (29%)	44.1 (13.3)	27.0 (2.4) (*n* = 19)	6 (33%) (*n* = 18)	1 (5%) (*n* = 19)	3.2 (5.5)	0(0%)/1(3%)/17(54%)/ 0(0%)/12(39%)/1(3%)

Abbreviations: BMI: Body Mass Index. CRP: C-reactive protein.

**Table 2:** The KP metabolites in the participating groups were given as mean (SD) and median (min; max).

Diagnosis	Trypt ng/mL	Kyn ng/mL	KA ng/mL	QA ng/mL	XA ng/mL
Obesity (before surgery, *n* = 139)	12,643 (2786) 12,439 (4692; 21,218)	489 (143) 472 (144; 1325)	14.3 (7.2) 12.5 (5.4; 68.4)	111 (45) 104 (47; 383)	5.3 (1.2) 5.2 (2.0; 9.2)
Obesity (after surgery, *n* = 95)	11,339 (1912) 11,589 (7195; 16,619)	378 (91) 361 (225; 641)	8.5 (3.9) 7.6 (1.9; 25.6)	84 (30) 80 (31; 191)	4.6 (0.9) 4.7 (2.9; 6.8)
Depression (*n* = 49)	14,106 (2677) 13,853 (9650; 21,192)	509 (149) 515 (240; 1047)	12.6 (5.8) 11.2 (4.7; 32.6)	101 (39) 92 (33; 207)	5.9 (1.1) 5.7 (3.8; 8.4)
Neurological (*n* = 31)	13,699 (3402) 13,038 (8019; 23,460)	461 (201) 391 (248; 1054)	12.1 (5.0) 10.8 (6.2; 25.1)	88 (59) 70 (40; 333)	5.7 (1.5) 5.8 (3.4; 9.9)
**Diagnosis**	**Kyn/Trypt** **× 1000**	**KA/Kyn** **× 1000**	**KA/QA** **× 1000**	**KA/XA**	**QA/XA**
Obesity (before surgery, *n* = 139)	39 (11) 37 (17; 90)	30 (12) 28 (13; 97)	138 (71) 119 (38; 475)	2.8 (1.3) 2.5 (1.0; 10.3)	21.9 (8.8) 20.2 (5.7; 57.9)
Obesity (after surgery, *n* = 95)	34 (7) 32 (21; 57)	22 (8) 21 (8; 51)	107 (52) 99 (31; 371)	1.8 (0.8) 1.7 (0.5; 5.6)	18.4 (6.3) 16.9 (8.8; 40.1)
Depression (*n* = 49)	36 (10) 35 (17; 62)	25 (10) 26 (11; 47)	137 (68) 123 (36; 380)	2.2 (1.0) 1.9 (0.8; 5.8)	17.4 (7.0) 15.8 (6.9; 40.4)
Neurological (*n* = 31)	34 (12) 31 (19; 78)	28 (7) 26 (17; 44)	154 (70) 145 (62; 367)	2.1 (0.8) 2.0 (1.2; 4.0)	15.5 (6.9) 14.1 (5.8; 37.4)

Abbreviations: KP: Kynurenine pathway. Trypt: Tryptophan. Kyn: Kynurenine. KA: Kynurenic acid. QA: Quinolinic acid. XA: Xanthurenic acid.

**Table 3:** The original KP metabolites in the three E-groups with comparisons between the groups. A linear mixed model adjusted for age, sex, diagnosis and point of time (before and after surgery for the obesity group) was used. The results are given as estimated marginal means with 95% confidence intervals and *p*-values for the overall comparisons.

KP Metabolites	E2 (*n* = 18) Mean (95% CI)	E3 (*n* = 120) Mean (95% CI)	E4 (*n* = 81) Mean (95% CI)	*p*-Values
Trypt (ng/mL)	13,396 (12,282;14,509)	13,266 (12,781;13,750)	13,075 (12,511;13,640)	0.806 0.696 *
Kyn (ng/mL)	479 (407;532)	474 (448;501)	442 (412;473)	0.242 0.439 *
KA (ng/mL)	12.3 (9.6;15.1)	12.4 (11.2;13.6)	11.1 (9.8;12.5)	0.313 0.358 *
QA (ng/mL)	90.9 (71.6;110.2)	102.0 (94.2;110.5)	90.2 (80.7;99.7)	0.101 0.201 *
XA (ng/mL)	5.6 (5.1;6.0)	5.5 (5.3;5.7)	5.4 (5.2;5.7)	0.842 0.758 *
Kyn/Trypt ratio × 1000	35 (31;40)	36 (34;38)	34 (32;37)	0.471 0.575 *
KA/Kyn ratio × 1000	27 (23;32)	26 (24;28)	25 (23;28)	0.702 0.698 *
KA/QA ratio × 1000	146 (116;175)	132 (120;147)	133 (118;147)	0.691 0.490 *
KA/XA ratio	2.3 (1.8;2.7)	2.3 (2.1;2.5)	2.1 (1.8;2.3)	0.337 0.541 *
QA/XA ratio	16.2 (13.0;19.4)	18.8 (17.4;20.1)	17.1 (15.5;18.7)	0.125 0.264 *

* Results of ln-transformed metabolites. Abbreviations: KP: Kynurenine pathway. Trypt: Tryptophan. Kyn: Kynurenine. KA: Kynurenic acid. QA: Quinolinic acid. XA: Xanthurenic acid.

**Table 4:** Pairwise differences between the KP metabolites and the E-groups were analysed with linear mixed models adjusted for age, sex, diagnosis and point of time (before and after bariatric surgery). The results are based on 10,000 bootstrap samples and reported as the regression coefficients (differences between the means) with 95% CI (percentile) and *p*-values. Significant *p*-values (defined as ≤ 0.01) are in boldfaced type.

KP Metabolites	E2 Compared with E3E4	E3 Compared with E2E4	E4 Compared with E2E3
Trypt (ng/mL)	207 (−425;786) *p* = 0.390	128 (−372;618) *p* = 0.519	−208 (−738;329) *p* = 0.327
Kyn (ng/mL)	8.1 (−29.7;44.3) *p* = 0.613;	26.9 (−1.2;48.8) *p* = 0.020;	−31.3 (−54.2;−3.2) ^1^ *p* = 0.008
KA (ng/mL)	0.45 (−1.1;2.2) *p* = 0.524	1.0 (−0.2;2.0) *p* = 0.056	−1.3 (−2.2;−0.1) *p* = 0.018
QA (ng/mL)	−6.5 (−15.2;2.4) *p* = 0.090	12.0 (3.5;18.6) ^2^ *p* = 0.005	−10.6 (−17.4;−2.2) *p* = 0.007
XA (ng/mL)	0.09 (−0.18;0.34) *p* = 0.382	0.04 (−0.17;0.25) *p* = 0.611	−0.08 (−0.30;0.15) *p* = 0.395
Kyn/Trypt ratio × 1000	−0.3 (−3.1;2.5) *p* = 0.765	1.6 (−0.4;3.0) *p* = 0.051	−1.6 (−3.0;0.4) *p* = 0.050
KA/Kyn ratio × 1000	1.4 (−1.6;4.6) *p* = 0.284	0.4 (−1.3;2.2) *p* = 0.537	−0.9 (−2.7;0.8) *p* = 0.202
KA/QA ratio × 1000	13.0 (−6.2;34.4) *p* = 0.125	−2.7 (−14.8;8.8) *p* = 0.594	−1.5 (−13.9;10.5) *p* = 0.772
KA/XA ratio	0.11 (−0.22;0.45) *p* = 0.439	0.16 (−0.04;0.34) *p* = 0.051	−0.21 (−0.39;−0.02) *p* = 0.011
QA/XA ratio	−1.9 (−3.5;−0.2) *p* = 0.008	1.8 (0.6;3.2) *p* = 0.001	−1.3 (−2.7;−0.0) *p* = 0.018

^1^ The differences in Kyn between ɛ3ɛ4 and ɛ4ɛ4 on one side and E2E3 were −40.1, (−67.3;−6.7) *p* = 0.001; and 64.6 (−70.2;226.4), *p* = 0.285, respectively. ^2^ The differences in QA between ɛ3ɛ3 and ɛ2ɛ4 on one side and E2E4 were 11.05 (3.95;16.38), *p* = 0.008 and 38.12 (22.16;50.69), *p* < 0.001, respectively. Abbreviations: KP: Kynurenine pathway. Trypt: Tryptophan. Kyn: Kynurenine. KA: Kynurenic acid. QA: Quinolinic acid. XA: Xanthurenic acid. Statistically significant *p*-values are in boldfaced types.

**Table 5:** Associations between Kyn and E4, and between QA and E3 analysed with linear mixed model regression adjusted for age, sex, BMI, diabetes, smoking, CRP, diagnosis and point of time relative to bariatric surgery. The results are shown with 10,000 bootstrap samples, 95%CIs (percentile) and *p*-values.

KP Metabolites	Age	Sex (Male)	BMI	Diabetes	Smoking	CRP	Comparisons between E-Groups
							E4 compared with E2E3
Kyn	4.10 (1.77;5.70) *p* < 0.001	−4.2 (−41.8;28.3) *p* = 0.774	2.2 (−4.0;7.8) *p* = 0.405	−33 (−76;20) *p* = 0.107	−16 (−99;53) *p* = 0.681	1.83 (−5.14;5.34) *p* = 0.574	−18.3 (−49.9;13.1) *p* = 0.163
							E3 compared with E2E4
QA	1.14 (0.59;1.61) *p* < 0.001	3.8 (−8.2;15.0) *p* = 0.470	0.5 (−0.8;2.5) *p* = 0.435	−10 (−26;3) *p* = 0.097	−11 (−21;−4) *p* = 0.046	1.26 (−0.32;2.81) *p* = 0.136	11.95 (2.4;19.6) *p* = 0.006

Abbreviations: KP: Kynurenine pathway. BMI: Body Mass Index. CRP: C-reactive protein. Kyn: Kynurenine. QA: Quinolinic acid. Statistically significant *p*-values are in boldfaced types.

## Data Availability

The raw data sets generated and analysed during the current study are not publicly available in order to protect participant confidentiality. Case report forms (CRFs) on paper are safely stored. The data were transferred to SPSS for statistical analyses, and the data files are stored by Innlandet Hospital Trust, Brumunddal, Norway, on a server dedicated to research. The security follows the rules given by The Norwegian Data Protection Authority, PO Box 8177 Dep. NO-0034 Oslo, Norway. The data are available on request to the corresponding author.
